# The Prevalence of Primary Angle Closure Glaucoma in Adult Asians: A Systematic Review and Meta-Analysis

**DOI:** 10.1371/journal.pone.0103222

**Published:** 2014-07-24

**Authors:** Jin-Wei Cheng, Ying Zong, You-Yan Zeng, Rui-Li Wei

**Affiliations:** 1 Department of Ophthalmology, Shanghai Changzheng Hospital, Second Military Medical University, Shanghai, China; 2 Department of Health Toxicology, Second Military Medical University, Shanghai, China; 3 School of Nursing, Second Military Medical University, Shanghai, China; Casey Eye Institute, United States of America

## Abstract

**Background:**

Primary angle closure glaucoma (PACG) is higher in Asians than Europeans and Africans, with over 80% of PACG worldwide in Asia. Previous estimates of PACG were based largely on early studies, mostly using inappropriate case definitions. Therefore, we did a systematic review and meta-analysis to estimate the prevalence of PACG in adult Asian populations and to quantify its association with age, gender, and region.

**Methods:**

All primary reports of population-based studies that reported the prevalence of PACG in adult Asian populations were identified. PACG case definition was compatible with the ISGEO definition. Twenty-nine population-based studies were included. The overall pooled prevalence estimates were calculated using a random effect model, and ethnicity-, age- and gender-specific pooled prevalence estimates were also calculated.

**Results:**

The overall pooled prevalence of PACG in those of adult Asians was 0.75% (95% CI, 0.58, 0.96). Ethnicity-specific pooled prevalence estimates were 0.97% (0.22, 4.27) in Middle East group, 0.66% (0.23, 1.86) in South East Asia group, 0.46% (0.32, 0.64) in India group, 1.10% (0.85, 1.44) in China group, and 1.19% (0.35, 3.98) in Japan group, respectively. Age-specific prevalence was 0.21% (0.12, 0.37) for those 40–49 years, 0.54% (0.34, 0.85) for those 50–59 years, 1.26% (0.93, 1.71) for those 60–69 years, and 2.32% (1.74, 3.08) for those 70 years or above. The overall female to male ratio of the PACG prevalence was 1.51∶1 (95% CI 1.01, 2.28).

**Conclusions:**

PACG affects approximately 0.75% adult Asians, increasing double per decade, and 60% of cases being female. The prevalence rates vary greatly by ethnic region.

## Introduction

Glaucoma is considered as the leading cause of irreversible blindness worldwide, with Asians accounting for approximately half of the world's glaucoma cases [1]. It also has been accepted that primary angle closure glaucoma (PACG) is higher in Asians than Europeans and Africans, with over 80% of those with PACG in Asia [1], [2]. Because PACG appears to cause blindness more frequently than primary open angle glaucoma (POAG), it is an important public health issue.

The current understanding of PACG in Asian populations is based largely on previous studies [1]–[3]. Early studies using the definitions of glaucoma based on intraocular pressure (IOP) reported that the prevalence of PACG in adults was 0.34% in Japan [4], 1.49% in Mongolia [5], 1.37% in China [6], and 1.18% in India [7], respectively. However, the earlier definitions of glaucoma are no longer accepted [8], and the prevalence rates reported in these earlier studies may not be accurate and comparable [9].

The International Society of Geographical & Epidemiological Ophthalmology (ISGEO) definition has demonstrated the general accepted classification for the diagnosis of glaucoma in population-based prevalence surveys [8]. However, the current understanding of PACG in Asians is based largely on studies using the earlier definitions of glaucoma, but not the ISGEO definition, which increasingly was seen as inadequate for both clinical and research purposes [1], [2], [9]. Recently, many population-based surveys of glaucoma in Asians using the ISGEO definition have been conducted. Because of the uncertainty surrounding the prevalence of PACG, this systematic review was to summarize the available population-based studies reporting prevalence values in Asians, to estimate an overall prevalence of PACG consistent with the ISGEO definition requiring structural and/or functional evidence of glaucomatous optic neuropathy.

## Materials and Methods

This meta-analysis was performed according to a predetermined protocol, and the methods used conformed to the Meta-analysis of Observational Studies in Epidemiology and the relevant aspects of the PRISMA statement [10], [11].

### Search Strategy

We used three methods to identify publications that reported the prevalence of PACG among Asian populations. First, we conducted a systematic search of the PubMed and EMBASE electronic databases from each database's inception date to February 10, 2014. Broad MeSH terms and keywords were used combining terms related to epidemiology (including MeSH search using exp prevalence*, and exp epidemiology*, and keyword search using words *prevalence*, *epidemiology*, and *incidence*), terms related to disease (including MeSH search using exp glaucoma*, and keyword search using words *glaucoma*), and terms related to population (including MeSH search using exp Asia*, and keyword search using words *Asia*, and *Asian*). Second, we hand-searched the reference lists of the relevant reviews, such as *Rudnicka 2006* [12], *Quigley 2006* [1], *Wong 2006* [2], *Zhou 2007* [3], and *Cheng 2013* [13]. Third, we consulted the reference lists of included articles to find additional studies.

### Study Selection

Published studies were included if they met the following inclusion criteria: (i) population-based, cross-sectional survey studies, with either random or consecutive sampling; (ii) adult Asian populations, customarily aged 40 years and older; (iii) a examination rate of the eligible population sample not less than 50%; (iv) PACG case definitions compatible with the current ISGEO definition based on structural and/or functional evidence of glaucomatous optic neuropathy in the presence of an occludable anterior chamber angle.

To determine study eligibility, three independent researchers screened the titles and abstracts of all search results, and all citations were classified into one of two categories: (i) relevant; (ii) irrelevant. The full articles of relevant citations were retrieved for further review to evaluate whether they met the inclusion criteria or not. Only eligible trials were assessed for methodological quality. Disagreements were resolved by consensus in both phases.

### Data Extraction

The following detailed information was extracted into a customized proforma: (i) study information (study name, publication year, citation, and study type), (ii) basic study data (geographical region, country of survey area, conditions in survey area, data collection year, sample size, and sociodemographic characteristics), (iii) quality-related data and outcome measures data (target population, sampling design, completeness of data/response rate, data collection, prevalence, definition and identification procedures for outcomes). Three reviewers independently carried out the data extraction, and inconsistencies were resolved by discussion with another independent reviewer.

### Risk of Bias Assessment

Two reviewers independently assessed the risk of bias for each included study, using a checklist developed from an existing tool assessing risk of bias in prevalence studies. The tool includes 10 items that assess measurement bias, selection bias, and bias related to the analysis (all rated as either high or low risk) and an overall assessment of risk of bias rated as either low, moderate, or high risk [14], [15]. To adapt to the needs of this meta-analysis, we also modified item 9 as “Were the screening process and assessing methods for the parameter of interest appropriate?” [16], [17].

Agreement was measured using kappa value as recommended by the Cochrane Handbook for Systematic Reviews of Interventions [18], and disagreement was resolved finally by discussion. Overall agreement between the reviewers was 93% with a kappa value of 0.76, indicating excellent agreement.

### Statistical Analysis

All statistical analyses were performed using Comprehensive Meta-Analysis software version 2.0 (Biostat, Englewood Cliffs, New Jersey) (http://www.meta-analysis.com). The primary outcome for each study was the prevalence proportion, calculated as the ratio of the number of individuals with PACG to the total number of study participants. The *I* ^2^ statistic was used to determine heterogeneity across studies, which quantify heterogeneity irrespective of the number of studies [19], [20]. The estimate and its 95% confidence intervals (CI) of overall proportion was calculated using the random effects model where heterogeneity was found [21], otherwise, the fixed effects model was used [22].

Ethnicity-specific pooled prevalence estimates of PACG were calculated, using a random effect model, which included the dominant ethnic group of five regions in Asia: Middle East, South East Asia, India, China, and Japan [1]. Age- and gender-specific pooled prevalence estimates of PACG were also calculated. A random-effect meta-regression model was built with ethnicity, age, and gender.

In addition, to attempt to control for potential methodologic heterogeneity, a random-effect regression model was also used to evaluate sources of variability in the overall pooled-prevalence estimate, such as urbanicity, the definition of occludable angle and the individual risk-of-bias items.

## Results

### Study Characteristics

**Figure 1:**
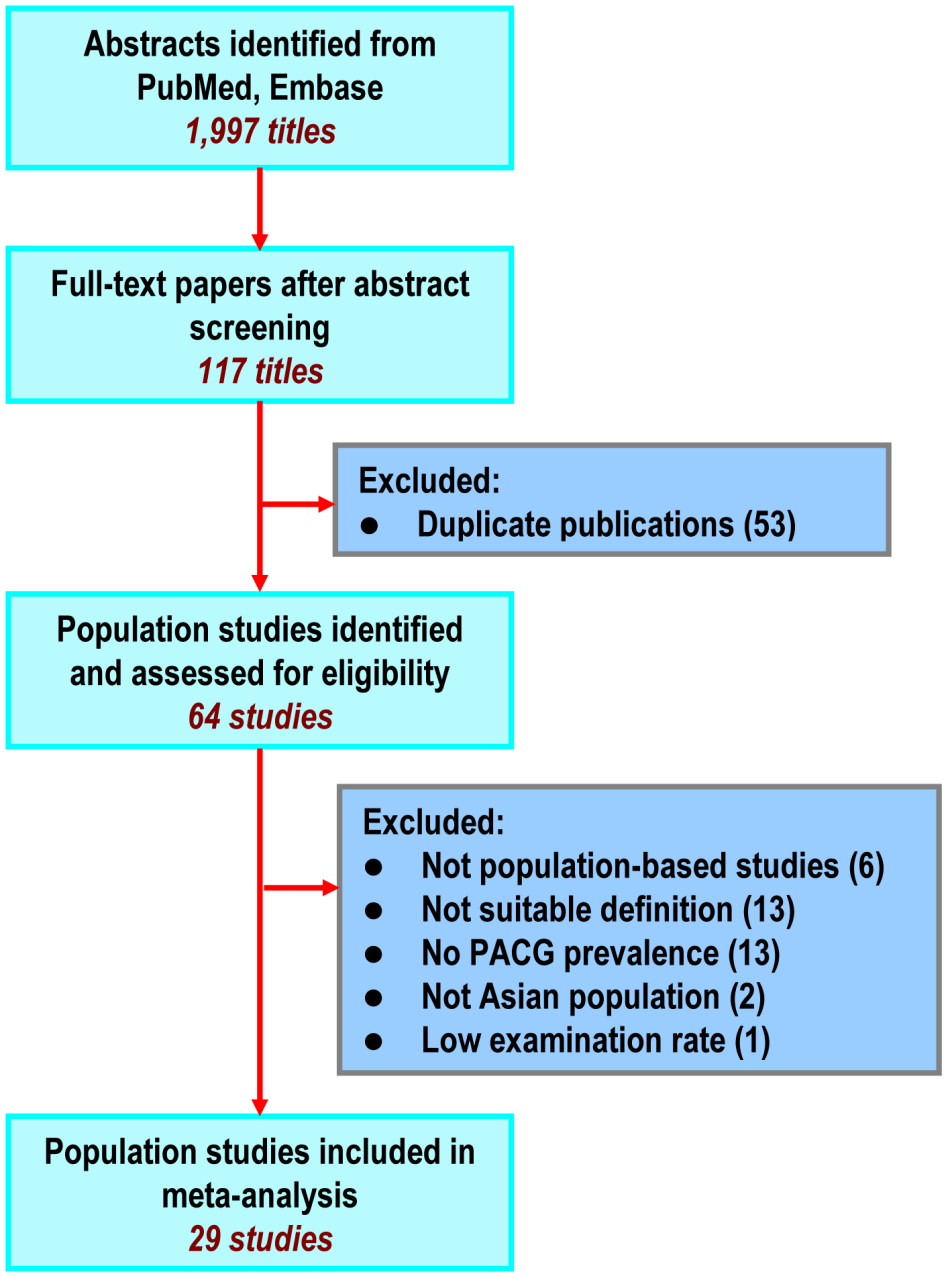
Flow Diagram of Study Selection. PACG: primary angle closure glaucoma

**Figure 1** shows the flow chart of the selection process used to identify relevant studies. We reviewed the full text of 117 articles from 1997 studies identified from the literature search, and 88 articles were excluded (**Appendix S1**). Twenty-nine population studies met all the inclusion criteria and were used to calculate the best evidence PACG prevalence estimates in adult Asian populations (**Table 1**) [23]–[51]. Seven studies (24%) were conducted in China, 5 (17%) in India, 3 (10%) in Singapore, 2 (7%) in Japan, Korea, and Nepal, and 1 (3%) in Bangladesh, Iran, Mongolia, Myanmar, Oman, Qatar, Thailand, and Sri Lanka. Fifteen studies (52%) were undertaken in rural, 6 (21%) in urban, and 8 (28%) in mixed populations. The age ranges of the studied populations were 30 years and over, with the majority of studies (n = 20, 69%) being 40 years and over, and the male portion of the populations ranged from 36% to 64%. Twenty-five studies (86%) used ISGEO definition for the diagnosis of PACG.

**Table 1:** Population Characteristics of the Studies Reported the Prevalence of Primary Angle Closure Glaucoma in Asians.

Study	Country	Urbanicity	Examined Year	Response (%)	Age Range (yrs)	N	Sex Ratio (M/F)	Case Definition	Angle Examination	Occludable Angle Definition	Adult PACG n (%)
Aravind Comprehensive Eye Survey [23]	India	Rural	1995–1997	93.0	≥40	5150	2836/2314	Angle + (GON ± GVFD)	Gonioscopy	Shaffer grade 0	26 (0.50)
Mongolia Eye Study [24]	Mongolia	Rural, urban	1995, 1997	95.4	≥40	1717	1007/710	ISGEO	Gonioscopy	270° ITM	28 (1.63)
Tanjong Pagar Eye Study [25]	Singapore	Urban	1997–1998	71.8	40–79	1232	557/670	ISGEO	Gonioscopy	270° ITM	14 (1.14)
Rom Klao Eye Study [26]	Thailand	Urban	1999	88.7	≥50	701	249/452	ISGEO	Gonioscopy	270° ITM	6 (0.86)
Dhaka Eye Study [27]	Bangladesh	Rural	1997–1998	65.9	≥35	2347	1120/1127	ISGEO	Gonioscopy	240° ITM	7 (0.30)
Tajimi Study [28]	Japan	Urban	2000–2001	78.1	≥40	3021	1334/1687	ISGEO	Gonioscopy	270° ITM	19 (0.63)
Shaanxi Rural Study [29]	China	Rural	2003	81.0	≥40	2835	1246/1587	Angle+IOP+(GVFD+/−GON)	Gonioscopy	Shaffer grade 0	31 (1.09)
West Bengal Glaucoma Study [30]	India	Rural	1998–1999	83.1	≥50	1324	611/658	ISGEO	Gonioscopy	240° ITM	3 (0.24)
Chennai Glaucoma Study [31]	India	Rural	2001–2004	80.2	≥40	3850	1710/2140	ISGEO	Gonioscopy	180° ITM	34 (0.88)
Liwan Eye Study [32]	China	Urban	2003–2004	75.3	≥50	1405	613/792	ISGEO	Gonioscopy	270° ITM	21 (1.53)
Meiktila Eye Study [33]	Myanmar	Rural	2005	83.7	≥40	2076	836/1240	ISGEO	Gonioscopy	270° ITM	52 (2.50)
Oman Eye Study [34]	Oman	Rural, urban	2005–2006	79.5	≥30	3324	1289/2035	Angle + (GON ± GVFD)	Gonioscopy	Shaffer grade 2	68 (2.05)
Singapore Malay Eye Study [35]	Singapore	Urban	2004–2006	78.7	≥40	3280	1576/1704	ISGEO	Gonioscopy	180° ITM	8 (0.24)
Kandy Eye Study [36]	Sri Lanka	Rural	2006–2007	79.9	≥40	1351	539/812	ISGEO	Gonioscopy	270° ITM	7 (0.57)
Sunsari Eye Study [37]	Nepal	Rural	2003–2004	80.0	≥40	1600	789/811	Angle+IOP+(GVFD+/−GON)	Gonioscopy	Shaffer grade 0	2 (0.13)
Andhra Pradesh Eye Disease Study [38]	India	Rural, urban	1996–2000	87.3	≥40	3724	1751/1973	ISGEO	Gonioscopy	180° ITM	35 (0.94)
Beijing Eye Study [39]	China	Rural, urban	2001	83.4	≥40	4315	1889/2412	ISGEO	Gonioscopy	270° ITM	44 (1.02)
Bin Eye Study [40]	China	Rural	2000	80.0	≥40	4956	2228/2728	ISGEO	Gonioscopy	270° ITM	78 (1.57)
Handan Eye Study [41]	China	Rural	2007	90.4	≥40	5480	2557/2923	ISGEO	Gonioscopy	180° ITM	30 (0.55)
Kailu Eye Study [42]	China	Rural	2009	87.4	≥40	5158	2299/2859	ISGEO	Gonioscopy	270° ITM	90 (1.74)
Sangju Eye Study [43]	Korea	Rural	-	60.0	≥50	671	264/407	ISGEO	Gonioscopy	270° ITM	2 (0.30)
Qatar Eye Study [44]	Qatar	Rural, urban	2009	97.7	≥40	3149	2015/1134	ISGEO	Gonioscopy	270° ITM	14 (0.44)
Namil Study [45]	Korea	Rural	2007–2008	79.5	≥40	1426	625/801	ISGEO	Gonioscopy	270° ITM	10 (0.70)
Bhaktapur Glaucoma Study [46]	Nepal	Rural, urban	2007–2009	83.4	≥40	3991	1819/2172	ISGEO	Gonioscopy	270° ITC	17 (0.43)
Kumejima Study [47]	Japan	Rural	2005–2006	81.2	≥40	3762	1833/1929	ISGEO	Gonioscopy	270° ITM	82 (2.18)
Yunnan Minority Eye Study [48]	China	Rural	2010	77.8	≥50	2133	769/1364	ISGEO	Gonioscopy	270° ITM	20 (0.94)
Central India Eye and Medical Study [49]	India	Rural	2006–2008	80.1	≥30	4711	2191/2520	ISGEO	Gonioscopy	270° ITM	14 (0.30)
Singapore Indian Eye Study [50]	Singapore	Urban	2007–2009	75.6	≥40	3400	1706/1694	ISGEO	Gonioscopy	180° ITM	6 (0.18)
Yazd Eye Study [51]	Iran	Rural, urban	2010–2011	90.4	≥40	1990	922/1068	ISGEO	Gonioscopy	270° ITM	7 (0.33)

GON: glaucomatous optic neuropathy; GVFD: glaucomatous visual field defect; IOP: intraocular pressure; ISGEO: International Society of Geographical & Epidemiological Ophthalmology.

ITM: invisible trabecular meshwork; ITC: iridotrabecular contact.

PACG: primary angle closure glaucoma.

### Risk of Bias

Overall, 22 studies (76%) were rated as having a low risk of bias, 7 (24%) were rated as having a moderate risk of bias (**Table 2**). High risk-of-bias ratings were most common for item I (national representativeness/target population), item IV (non-response bias), item VI (case definition), and item VII (study instrument).

**Table 2:** Risk of Bias of the Studies Reported the Prevalence of Primary Angle Closure Glaucoma in Asians.

Study	I	II	III	IV	V	VI	VII	VIII	IX	X	Overall
Aravind Comprehensive Eye Survey [23]	High	Low	Low	Low	Low	High	Low	Low	Low	Low	Moderate
Mongolia Eye Study [24]	High	Low	Low	Low	Low	Low	Low	Low	Low	Low	Low
Tanjong Pagar Eye Study [25]	High	Low	Low	High	Low	Low	Low	Low	Low	Low	Moderate
Rom Klao Eye Study [26]	High	Low	Low	Low	Low	Low	Low	Low	Low	Low	Low
Dhaka Eye Study [27]	High	Low	Low	High	Low	Low	Low	Low	Low	Low	Moderate
Tajimi Study [28]	High	Low	Low	Low	Low	Low	Low	Low	Low	Low	Low
Shaanxi Rural Study [29]	High	Low	Low	Low	Low	High	High	Low	Low	Low	Moderate
West Bengal Glaucoma Study [30]	High	Low	Low	Low	Low	Low	Low	Low	Low	Low	Low
Chennai Glaucoma Study [31]	High	Low	Low	Low	Low	Low	Low	Low	Low	Low	Low
Liwan Eye Study [32]	High	Low	Low	Low	Low	Low	Low	Low	Low	Low	Low
Meiktila Eye Study [33]	High	Low	Low	Low	Low	Low	Low	Low	Low	Low	Low
Oman Eye Study [34]	Low	Low	Low	Low	Low	High	Low	Low	Low	Low	Moderate
Singapore Malay Eye Study [35]	Low	Low	Low	Low	Low	Low	Low	Low	Low	Low	Low
Kandy Eye Study [36]	High	Low	Low	Low	Low	Low	Low	Low	Low	Low	Low
Sunsari Eye Study [37]	High	Low	Low	Low	Low	High	Low	Low	Low	Low	Moderate
Andhra Pradesh Eye Disease Study [38]	High	Low	Low	Low	Low	Low	Low	Low	Low	Low	Low
Beijing Eye Study [39]	High	Low	Low	Low	Low	Low	Low	Low	Low	Low	Low
Bin Eye Study [40]	High	Low	Low	Low	Low	Low	Low	Low	Low	Low	Low
Handan Eye Study [41]	High	Low	Low	Low	Low	Low	Low	Low	Low	Low	Low
Kailu Eye Study [42]	High	Low	Low	Low	Low	Low	Low	Low	Low	Low	Low
Sangju Eye Study [43]	High	Low	Low	High	Low	Low	Low	Low	Low	Low	Moderate
Qatar Eye Study [44]	Low	Low	Low	Low	Low	Low	Low	Low	Low	Low	Low
Namil Study [45]	High	Low	Low	Low	Low	Low	Low	Low	Low	Low	Low
Bhaktapur Glaucoma Study [46]	High	Low	Low	Low	Low	Low	Low	Low	Low	Low	Low
Kumejima Study [47]	High	Low	Low	Low	Low	Low	Low	Low	Low	Low	Low
Yunnan Minority Eye Study [48]	High	Low	Low	Low	Low	Low	Low	Low	Low	Low	Low
Central India Eye and Medical Study [49]	High	Low	Low	Low	Low	Low	Low	Low	Low	Low	Low
Singapore Indian Eye Study [50]	Low	Low	Low	Low	Low	Low	Low	Low	Low	Low	Low
Yazd Eye Study [51]	High	Low	Low	Low	Low	Low	Low	Low	Low	Low	Low
I	Was the study's target population a close representation of the national population in relation to relevant variables, e.g., age, sex, occupation?										
II	Was the sampling frame a true or close representation of the target population?										
III	Was some form of random selection used to select the sample, OR, was a census undertaken?										
IV	Was the likelihood of non-response bias minimal?										
V	Were data collected directly from the subjects (as opposed to a proxy)?										
VI	Was an acceptable case definition used in the study?										
VII	Had the study instrument that measured the parameter of interest (e.g., prevalence of PACG) been tested for reliability and validity (if necessary)?										
VIII	Was the same mode of data collection used for all subjects?										
IX	Were the screening process and assessing methods for the parameter of interest appropriate?										
X	Were the numerator(s) and denominator(s) for the parameter of interest appropriate?										

### Meta-Analysis

**Figure 2:**
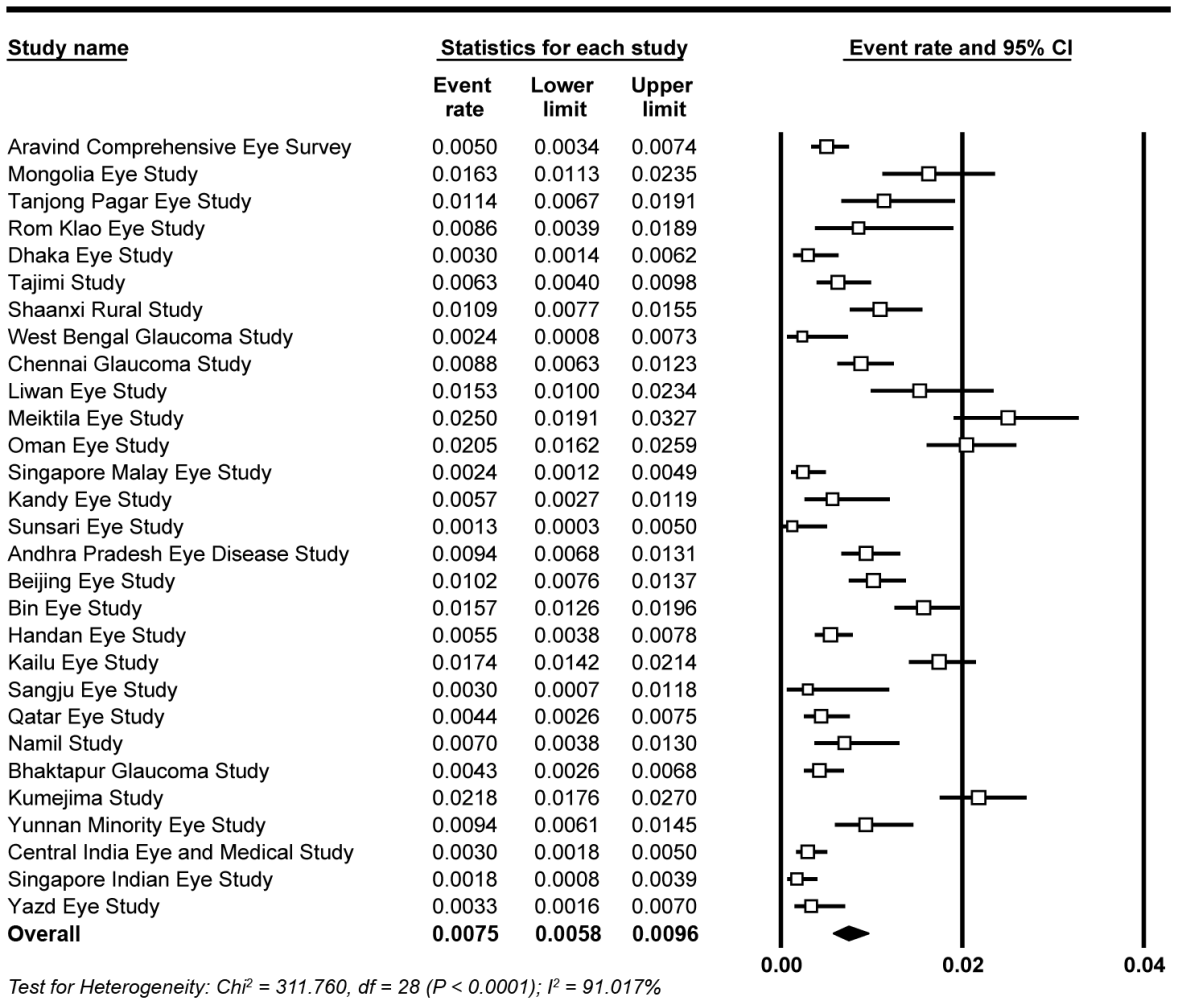
The forest plot of the prevalence of primary angle closure glaucoma.

The prevalence of PACG reported in the included studies varied from 0.13% to 2.50% in adult Asian populations (**Figure 2**). The heterogeneity in the prevalence of PACG was statistically significant and substantial in considerable. The overall random-effects estimate of the prevalence of PACG in adult Asian populations was 0.75% (95% CI, 0.58, 0.96).

**Figure 3:**
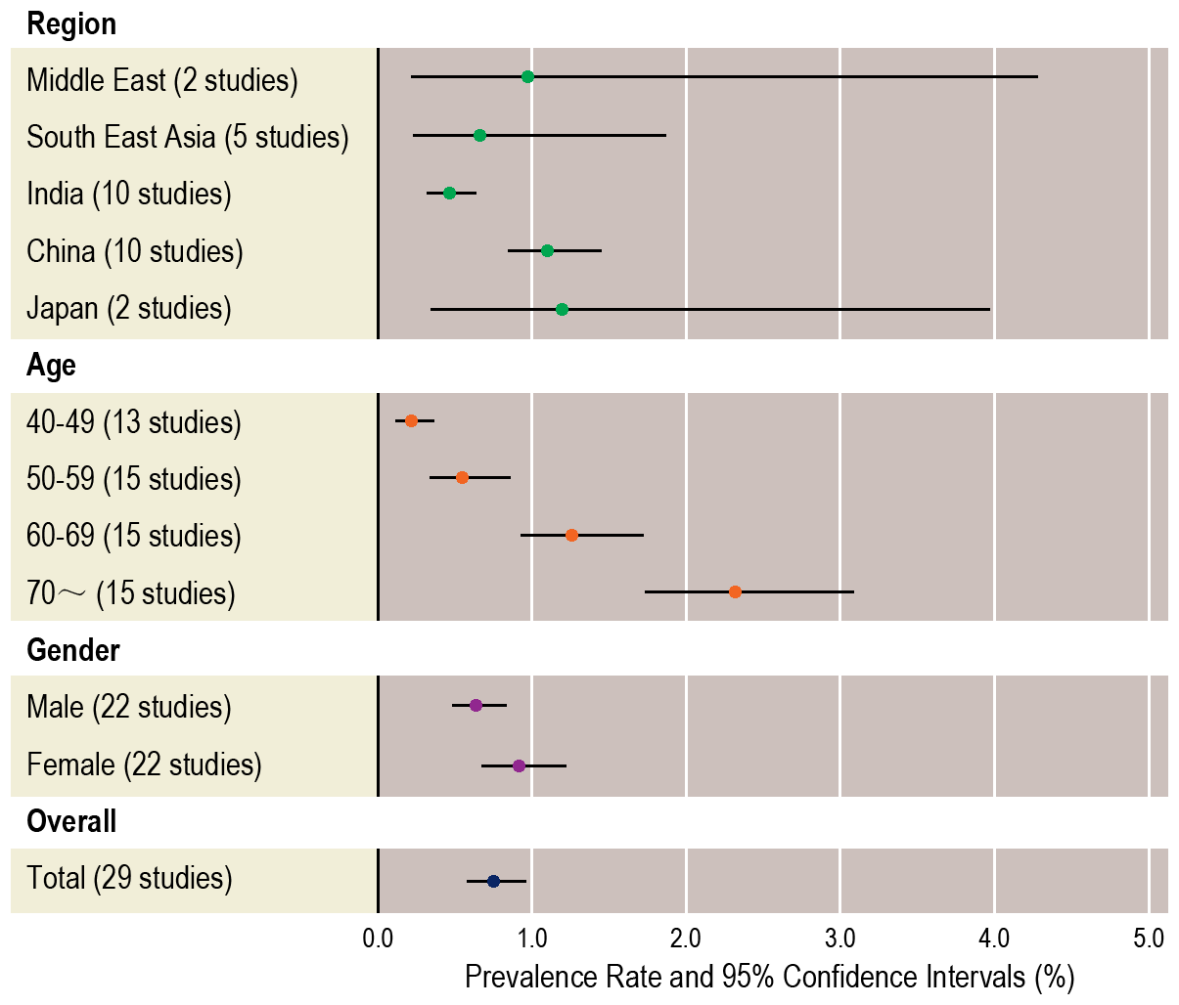
Ethnicity-, age- and gender-specific pooled prevalence rates of primary angle closure glaucoma.

Ethnicity-specific pooled prevalence estimates of PACG of five Asian regions are shown in Figure 3. The pooled prevalence estimates of PACG were 0.97% (95% CI, 0.22, 4.27) in Middle East group, 0.66% (0.23, 1.86) in South East Asia group, 0.46% (0.32, 0.64) in India group, 1.10% (0.85, 1.44) in China group, and 1.19% (0.35, 3.98) in Japan group, respectively. The meta-regression analyses showed there was a strong association of prevalence with ethnic group (β = 0.27, *P* = 0.009).

Fifteen studies reported age-specific prevalence of PACG, and twenty-two studies reported gender-specific prevalence. The age-specific prevalence was 0.21% (95% CI, 0.12, 0.37) for those 40–49 years old, 0.54% (0.34, 0.85) for those 50–59 years old, 1.26% (0.93, 1.71) for those 60–69 years old, and 2.32% (1.74, 3.08) for those 70 years old or above (**Figure 3**). Meta-regression analysis showed a high prevalence rate was strongly associated with an older age of sample (β = 0.74, *P*<0.0001). The pooled prevalence was 0.63% (0.49, 0.82) for male and 0.91% (0.68, 1.21) for female (**Figure 3**). Meta-regression analyses showed a strong association between a high prevalence rate and a higher proportion of female gender (β = 0.41, *P* = 0.047), and the overall female to male ratio of the PACG prevalence was 1.51∶1 (95% CI 1.01, 2.28).

The meta-regression analyses showed there was no association of the prevalence rate with urbanicity (β = −0.17, *P* = 0.524). The prevalence of PACG was also not associated with the definition of occludable angle (β = −0.05, *P* = 0.717). For risk-of-bias items, the prevalence rate was not associated with a high risk of bias for item I (β = 0.48, *P* = 0.217), item IV (β = −0.40, *P* = 0.391), item VI (β = 0.06, *P* = 0.882), and item VII (β = 0.77, *P* = 0.112).

## Discussion

This comprehensive systematic review was conducted to investigate the prevalence of PACG in Asian, and to understand the reasons of estimate variability. The findings showed that for those of adult Asian populations, 0.75% were estimated to have PACG. This systematic review also quantified the variability in the prevalence of PACG for age, gender and ethnic group. The rate of PACG prevalence increased with age, approximately double per decade. PACG prevalence in women was approximately 1.5 times that in men. There was a strong variability of PACG prevalence rates by ethnic group.

It has been established that the prevalence of glaucoma varies significantly by region [1], [9], [12], [52]. On the basis of the findings from this systematic review, there was also significant ethnic variation in the prevalence of PACG among five Asian regions. The highest prevalence rates of PACG were reported in Japan (1.19%) and China (1.10%), followed by Middle East (0.97%), South East Asia (0.66%), and India (0.46%). A recent systematic review found that the prevalence of PACG in those 40 years or more in European derived populations is 0.4% (95% CI 0.3% to 0.5%) [53]. Therefore, the prevalence of PACG in Asians, especially in East Asians and South East Asians, is higher than those in Europeans. However, the findings should be interpreted with caution, especially for the Japan, Middle East and South East Asia groups, because of the very wide confidence interval of prevalence rates, and the significantly large heterogeneity across included studies.

The pooled prevalence of PACG in five Asian ethnic group from the present review was inconsistent with the results reported in the previous reviews [1], [13]. In the previous reviews [1], [13], the prevalence of PACG was over-diagnosed in South East Asia, India, and China regions, and under-diagnosed in Middle East and Japan regions. Interpretation of the over- and under-diagnosis of PACG prevalence values is complicated by the inappropriate case definitions used in some studies diagnosing PACG, especially those based only on a narrow anterior chamber angle with raised IOP [13].

An appropriate case definition is the keystone of epidemiological research, and the ISGEO definition has commonly been accepted since it was published [1]. A consensus definition of an “occludable” angle in which the posterior (usually pigmented) trabecular meshwork is seen for less than 90°of angle circumference has come into common usage to indicate the anatomical predisposition to angle closure [1], [54], [55]. However, the definition of an “occludable” angle excluded around half of all participants who have primary peripheral anterior synechiae (PAS) [54]. Although a slightly more liberal threshold, 180 degrees of iridotrabecular contact (ITC), was used in many population studies, it is still likely to exclude many people who have primary PAS. Therefore, the most widely used epidemiological definition of an “occludable” angle, 180–270 degrees of ITC, is too stringent. The traditional view that primary angle closure becomes a significant possibility in the iridotrabecular angle of 20 degrees probably represents the most inclusive of approaches [54], [55]. In addition, gonioscopy using visible light probably under-detects cases where ITC is occurring [55]. Although the results of this present review showed no association between the prevalence of PACG and the definition of occludable angle, in future, the definition of an “occludable angle” used in epidemiological studies of glaucoma still should be reconsidered.

There are several limitations of this systematic review should be discussed. First, similar to most systematic reviews, a potential limitation is the publication bias. We attempted to avoid the potential for publication bias by conducting an extensive search. However, the studies published in languages other than English probably was missed. Second, available studies were from only 14 countries. Thus, more population-based studies should be required to estimates the whole prevalence in Asian populations. Third, the diagnostic criteria for glaucoma and occludable angle also differed among studies. Although no association between the prevalence and case definitions was found, the expanding definition of an ‘occludable’ angle will allow for better consideration of this possibility through research and clinical practice [55].

Nevertheless, this systematic review provides a current evidence-based estimate of PACG prevalence in Asian populations. In the past, the number of PACG worldwide probably was misestimated. PACG affects approximately 0.75% adult Asian populations, and the prevalence rates vary greatly by ethnic region. The findings of this present systematic review provide benefit to estimate the burden of PACG in Asia.

## Supporting Information

Appendix S1 **The excluded articles and the reason for exclusion.**
(PDF)

Checklist S1 **PRISMA Checklist.**
(DOC)
