# Supplementary material for: The Prevalence of Primary Angle Closure Glaucoma in Adult Asians: A Systematic Review and Meta-Analysis
Source: PLoS One. 2014 Jul 24;9(7):e103222. doi: 10.1371/journal.pone.0103222 (PMC4110010; doi:10.1371/journal.pone.0103222)
Supplement: Appendix S1 — The excluded articles and the reason for exclusion. (PDF) [file pone.0103222.s001.pdf]

Appendix S1. The excluded articles and the reason for exclusion

Article	Reason for exclusion
Awasthi 1975 [1]	Not report the prevalence of PACG
Jain 1983 [2]	Not report the prevalence of PACG
Leydhecker 1984 [3]	Hospital-based study, but not population-based study
Hu 1989 [4]	Not use the ISGEO definition, but based on IOP
Mason 1989 [5]	Latin American population, but not Asian population
Zhao 1990 [6]	Not use the ISGEO definition, but based on IOP
Shiose 1991 [7]	Not use the ISGEO definition, but based on IOP
Rauf 1994 [8]	Not report the prevalence of PACG
Congdon 1995 [9]	Not use the ISGEO definition, but based on IOP; low examination rate
Gao 1995 [10]	Not use the ISGEO definition, but based on IOP
Yu 1995 (1) [11]	Not use the ISGEO definition, but based on IOP
Yu 1995 (2) [12]	Duplicate publication of the excluded study: Yu 1995 (1)
Congdon 1996 [13]	Duplicate publication of the excluded study: Congdon 1995
Foster 1996 [14]	Duplicate publication of the included study: Mongolia Eye Study
Gray 1996 [15]	Not report the prevalence of PACG
Congdon 1997 [16]	Duplicate publication of the excluded study: Congdon 1995
Seah 1997 [17]	Not use the ISGEO definition, but based on IOP
Jacob 1998 [18]	Not use the ISGEO definition, but based on IOP
Sim 1998 [19]	Low examination rate
Sim 1999 [20]	Duplicate publication of the excluded study: Sim 1998
Dandona 2000 (1) [21]	Duplicate publication of the included study: Andhra Pradesh Eye Disease Study
Dandona 2000 (2) [22]	Duplicate publication of the included study: Andhra Pradesh Eye Disease Study
Thomas 2001 [23]	Duplicate publication of the included study: Andhra Pradesh Eye Disease Study
Lai 2001 [24]	Prospective study, but not population-based study
Lau 2002 [25]	Not report the prevalence of PACG
Metheetrairut 2002 [26]	Not use the ISGEO definition, but based on IOP
Zhao 2002 [27]	Not use the ISGEO definition, but based on IOP
Arvind 2003 [28]	Duplicate publication of the included study: Chennai Glaucoma Study
Foster 2003 [29]	Duplicate publication of the included study: Tanjong Pagar Eye Study
George 2003 [30]	Duplicate publication of the included study: Chennai Glaucoma Study
Nolan 2003 [31]	Randomized controlled trial, but not population-based study
Thomas 2003 (1) [32]	Duplicate publication of the included study: Andhra Pradesh Eye Disease Study
Thomas 2003 (2) [33]	Duplicate publication of the included study: Andhra Pradesh Eye Disease Study
Iwase 2004 [34]	Duplicate publication of the included study: Tajimi Study
Xu 2004 [35]	Duplicate publication of the included study: Beijing Eye Study

Article	Reason for exclusion
Aung 2005 [36]	Duplicate publication of the included study: Tanjong Pagar Eye Study
Bai 2005 (1) [37]	Duplicate publication of the included study: Shaanxi Rural Study
Bai 2005 (2) [38]	Duplicate publication of the included study: Shaanxi Rural Study
Ren 2005 [39]	Duplicate publication of the included study: Shaanxi Rural Study
Vijaya 2005 [40]	Duplicate publication of the included study: Chennai Glaucoma Study
Xu 2005 [41]	Duplicate publication of the included study: Beijing Eye Study
Nolan 2006 [42]	Duplicate publication of the included study: Mongolia Eye Study
Suzuki 2006 [43]	Duplicate publication of the included study: Tajimi Study
Vijaya 2006 [44]	Duplicate publication of the included study: Chennai Glaucoma Study
Casson 2007 (1) [45]	Duplicate publication of the included study: Meiktila Eye Study
Casson 2007 (2) [46]	Duplicate publication of the included study: Meiktila Eye Study
Xu 2007 [47]	Duplicate publication of the included study: Beijing Eye Study
Yuan 2007 [48]	Not use the ISGEO definition, but based on IOP
Hu 2008 [49]	Hospital-based study, but not population-based study
Khandekar 2008 [50]	Duplicate publication of the included study: Oman Eye Study
Palimkar 2008 [51]	Not use the ISGEO definition, but based on IOP
Vijaya 2008 [52]	Duplicate publication of the included study: Chennai Glaucoma Study
Zhang 2008 [53]	Duplicate publication of the included study: Shaanxi Rural Study
Casson 2009 [54]	Duplicate publication of the included study: Meiktila Eye Study
Haq 2009 [55]	Not report the prevalence of PACG
Wang 2019 [56]	Duplicate publication of the included study: Beijing Eye Study
Garudadri 2010 [57]	Duplicate publication of the included study: Andhra Pradesh Eye Disease Study
Huang 2010 [58]	Not report the prevalence of PACG
Nongpiur 2010 [59]	Not report the prevalence of PACG
Perera 2010 [60]	Duplicate publication of the included study: Singapore Malay Eye Study
Sia 2010 [61]	Duplicate publication of the included study: Kandy Eye Study
Zheng 2010 (1) [62]	Duplicate publication of the included study: Singapore Malay Eye Study
Zheng 2010 (2) [63]	Duplicate publication of the included study: Singapore Malay Eye Study
Hsu 2011 [64]	Not report the prevalence of PACG
Ishikawa 2011 [65]	Not population-based study
Kim 2011 [66]	Duplicate publication of the included study: Namil Study
Liang 2011 [67]	Duplicate publication of the included study: Handan Eye Study
Thapa 2011 [68]	Duplicate publication of the included study: Bhaktapur Glaucoma Study
Wang 2011 [69]	Duplicate publication of the included study: Liwan Eye Study
Yoon 2011 [70]	Not use the ISGEO definition, but based on IOP
Chung 2012 [71]	Not report the prevalence of PACG

Article	Reason for exclusion
Foo 2012 [72]	Not report the prevalence of PACG
Kim 2012 [73]	Duplicate publication of the included study: Namil Study
Sng 2012 [74]	Duplicate publication of the excluded study: Foo 2012
Suh 2012 [75]	Duplicate publication of the included study: Namil Study
Sun 2012 [76]	Duplicate publication of the included study: Bin Eye Study
Wang 2012 (1) [77]	Duplicate publication of the included study: Beijing Eye Study
Wang 2012 (2) [78]	Duplicate publication of the included study: Beijing Eye Study
Xu 2012 [79]	Not report the prevalence of PACG
Zhou 2012 [80]	Duplicate publication of the included study: Handan Eye Study
Choudhari 2013 [81]	Duplicate publication of the included study: Chennai Glaucoma Study
Katibeh 2013 [82]	Duplicate publication of the included study: Yazd Eye Study
Levkovitch-Verbin 2013 [83]	Retrospective study, but not population-based study
Pan 2013 [84]	Duplicate publication of the included study: Singapore Indian Eye Study
Rauf 2013 [85]	British Asian population, but not Asian population
Thapa 2013 [86]	Duplicate publication of the included study: Bhaktapur Glaucoma Study
Wang 2013 [87]	Not report the prevalence of PACG
Wu 2013 [88]	Duplicate publication of the included study: Singapore Malay Eye Study

PACG: primary angle closure glaucoma; IOP: intraocular pressure; ISGEO: International Society of Geographical & Epidemiological Ophthalmology.

References

1. Awasthi P, Sarbhai KP, Banerjee SC, Maheshwari BB (1975) Prevalence study of glaucoma in rural areas. Indian J Ophthalmol 23:1-5.
2. Jain MR, Modi R (1983) Survey of chronic simple glaucoma in the rural population of India (Udaipur) above the age group of 30 years. Indian J Ophthalmol 31: 656-657.
3. Leydhecker W (1984) A glaucoma survey in South India. Doc Ophthalmol 57: 357-359.
4. Hu CN (1989) An epidemiologic study of glaucoma in Shunyi County, Beijing. Zhonghua Yan Ke Za Zhi 25: 115-119.
5. Mason RP, Kosoko O, Wilson MR, Martone JF, Cowan CL Jr, et al. (1989) National survey of the prevalence and risk factors of glaucoma in St. Lucia, West Indies. Part I. Prevalence findings. Ophthalmology 96: 1363-1368.
6. Zhao JL (1990) An epidemiological survey of primary angle-closure glaucoma (PACG) in Tibet. Zhonghua Yan Ke Za Zhi 26: 47-50.
7. Shiose Y, Kitazawa Y, Tsukahara S, Akamatsu T, Mizokami K, et al. (1991) Epidemiology of glaucoma in Japan--a nationwide glaucoma survey. Jpn J Ophthalmol 35: 133-155.
8. Rauf A, Ong PS, Pearson RV, Wormald RP (1994) A pilot study into the prevalence of ophthalmic disease in the Indian population of Southall. J R Soc Med 87: 78–79.
9. Congdon NG, Quigley HA, Hung PT, Wang TH, Ho TC, et al. (1995) Impact of age, various forms of cataract, and visual acuity on whole-field scotopic sensitivity screening for glaucoma in rural

Taiwan. Arch Ophthalmol 113: 1138-1143.
10. Gao Z (1995) An epidemiologic study of glaucoma in Tongcheng county, Anhui province. Zhonghua Yan Ke Za Zhi 31: 149-151.
11. Yu Q, Xu J, Zhu S (1995) An epidemiological survey of primary angle-closure glaucoma in Doumen county Guangdong. Zhonghua Yan Ke Za Zhi 31: 118-121.
12. Yu Q, Xu J, Zhu S, Liu Q (1995) Epidemiological survey of primary angle-closure glaucoma in Doumen. Yan Ke Xue Bao 11: 5-8.
13. Congdon NG, Quigley HA, Hung PT, Wang TH, Ho TC (1996) Screening techniques for angle-closure glaucoma in rural Taiwan. Acta Ophthalmol Scand 74: 113-119.
14. Foster PJ, Baasanhu J, Alsbirk PH, Munkhbayar D, Uranchimeg D, et al. (1996) Glaucoma in Mongolia. A population-based survey in Hövsgöl province, northern Mongolia. Arch Ophthalmol 114: 1235-1241.
15. Gray PJ (1996) The prevalence of eye disease in elderly Bengalis in Tower Hamlets. J R Soc Med 89: 23-26.
16. Congdon NG, Youlin Q, Quigley H, Hung PT, Wang TH, et al. (1997) Biometry and primary angle-closure glaucoma among Chinese, white, and black populations. Ophthalmology 104: 1489-1495.
17. Seah SK, Foster PJ, Chew PT, Jap A, Oen F, et al. (1997) Incidence of acute primary angle-closure glaucoma in Singapore. An island-wide survey. Arch Ophthalmol 115: 1436-1440.
18. Jacob A, Thomas R, Koshi SP, Braganza A, Muliyil J (1998) Prevalence of primary glaucoma in an urban south Indian population. Indian J Ophthalmol 46: 81-86.
19. Sim DH, Goh LG, Ho T (1998) Glaucoma pattern amongst the elderly Chinese in Singapore. Ann Acad Med Singapore 27: 819-823.
20. Sim DH, Goh LG (1999) Screening for glaucoma in the Chinese elderly population in Singapore. Singapore Med J 40: 644-647.
21. Dandona L, Dandona R, Mandal P, Srinivas M, John RK, et al. (2000) Angle-closure glaucoma in an urban population in southern India. The Andhra Pradesh eye disease study. Ophthalmology 107: 1710-1716.
22. Dandona L, Dandona R, Srinivas M, Mandal P, John RK, et al. (2000) Open-angle glaucoma in an urban population in southern India: the Andhra Pradesh eye disease study. Ophthalmology 107: 1702-1709.
23. Thomas R, Muliyil J, George R (2001) Glaucoma in southern India. Ophthalmology 108: 1173-1175.
24. Lai JS, Liu DT, Tham CC, Li RT, Lam DS (2001) Epidemiology of acute primary angle-closure glaucoma in the Hong Kong Chinese population: prospective study. Hong Kong Med J 7:118-123.
25. Lau JT, Lee V, Fan D, Lau M, Michon J (2002) Knowledge about cataract, glaucoma, and age related macular degeneration in the Hong Kong Chinese population. Br J Ophthalmol 86: 1080-1084.
26. Metheetrairut A, Singalavanija A, Ruangvaravate N, Tuchinda R (2002) Evaluation of screening tests and prevalence of glaucoma: integrated health research program for the Thai elderly. J Med Assoc Thai 85: 147-153.
27. Zhao J, Sui R, Jia L, Ellwein LB (2002) Prevalence of glaucoma and normal intraocular pressure among adults aged 50 years or above in Shunyi county of Beijing. Zhonghua Yan Ke Za Zhi 38: 335-339.

28. Arvind H, Paul PG, Raju P, Baskaran M, George R, et al. (2003) Methods and design of the Chennai Glaucoma Study. Ophthalmic Epidemiol 10: 337-348.
29. Foster PJ, Machin D, Wong TY, Ng TP, Kirwan JF, et al. (2003) Determinants of intraocular pressure and its association with glaucomatous optic neuropathy in Chinese Singaporeans: the Tanjong Pagar Study. Invest Ophthalmol Vis Sci 44: 3885-3891.
30. George R, Paul PG, Baskaran M, Ramesh SV, Raju P, et al. (2003) Ocular biometry in occludable angles and angle closure glaucoma: a population based survey. Br J Ophthalmol 87: 399-402.
31. Nolan WP, Baasanhu J, Undraa A, Uranchimeg D, Ganzorig S, et al. (2003) Screening for primary angle closure in Mongolia: a randomised controlled trial to determine whether screening and prophylactic treatment will reduce the incidence of primary angle closure glaucoma in an east Asian population. Br J Ophthalmol 87: 271-274.
32. Thomas R, George R, Parikh R, Muliyil J, Jacob A (2003) Five year risk of progression of primary angle closure suspects to primary angle closure: a population based study. Br J Ophthalmol 87: 450-454.
33. Thomas R, Parikh R, Muliyil J, Kumar RS (2003) Five-year risk of progression of primary angle closure to primary angle closure glaucoma: a population-based study. Acta Ophthalmol Scand 81: 480-485.
34. Iwase A, Suzuki Y, Araie M, Yamamoto T, Abe H, et al. (2004) The prevalence of primary open-angle glaucoma in Japanese: the Tajimi Study. Ophthalmology 111: 1641-1648.
35. Xu L, Chen JH, Li JJ, Luo L, Yang H, et al. (2004) The prevalence and its screening methods of primary open angle glaucoma in defined population-based study of rural and urban in Beijing. Zhonghua Yan Ke Za Zhi 40: 726-732.
36. Aung T, Nolan WP, Machin D, Seah SK, Baasanhu J, et al. (2005) Anterior chamber depth and the risk of primary angle closure in 2 East Asian populations. Arch Ophthalmol 123:527-532.
37. Bai ZL, Ren BC, He Y, Yang JG, Chen L, et al. (2005) Epidemiology of primary open angle glaucoma in a rural population in Shaanxi Province of China. Guo Ji Yan Ke Za Zhi 5: 864-871.
38. Bai ZL, Ren BC, Yang JG, He Y, Chen L, et al. (2005) Systemic blood pressure, intraocular pressure and primary open-glaucoma: A population-based study in Shaanxi Province of China. Guo Ji Yan Ke Za Zhi 5: 1122-1127.
39. Ren BC, He Y, Chen L, Yang JG, Sun NX (2005) Epidemiology of glaucoma in a rural population in Shaanxi Province. Guo Ji Yan Ke Za Zhi 5: 1037-1042.
40. Vijaya L, George R, Paul PG, Baskaran M, Arvind H, et al. (2005) Prevalence of open-angle glaucoma in a rural south Indian population. Invest Ophthalmol Vis Sci 46: 4461-4467.
41. Xu L, Zhang L, Xia CR, Li JJ, Hu LN, et al. (2005) The prevalence and its effective factors of primary angle-closure glaucoma in defined populations of rural and urban in Beijing. Zhonghua Yan Ke Za Zhi 41: 8-14.
42. Nolan WP (2006) Primary angle closure glaucoma in East Asia: An overview of the Mongolia-based research programme. Asian J Ophthalmol 8: 6-12.
43. Suzuki Y, Iwase A, Araie M, Yamamoto T, Abe H, et al. (2006) Risk factors for open-angle glaucoma in a Japanese population: the Tajimi Study. Ophthalmology 113: 1613-1617.
44. Vijaya L, George R, Arvind H, Baskaran M, Paul PG, et al. (2006) Prevalence of angle-closure disease in a rural southern Indian population. Arch Ophthalmol 124: 403-409.
45. Casson RJ, Gupta A, Newland HS, McGovern S, Muecke J, et al. (2007) Risk factors for primary open-angle glaucoma in a Burmese population: the Meiktila Eye Study. Clin Experiment

Ophthalmol 35: 739-744.
46. Casson RJ, Newland HS, Muecke J, McGovern S, Abraham LM, et al. (2007) Gonioscopy findings and prevalence of occludable angles in a Burmese population: the Meiktila Eye Study. Br J Ophthalmol 91: 856-859.
47. Xu L, Wang Y, Wang S, Wang Y, Jonas JB (2007) High myopia and glaucoma susceptibility the Beijing Eye Study. Ophthalmology 114: 216-220.
48. Yuan HP, Yu H, Xiao Z, Shao ZB, Zhang XL, et al. (2007) The prevalence of primary angle-closure glaucoma and its causes in rural area of Shuangyang district in Changchun, Jilin province. Zhonghua Yan Ke Za Zhi 43: 775-778.
49. Hu CC, Lin HC, Chen CS (2008) A 7-year population study of primary angle closure glaucoma admissions and climate in Taiwan. Ophthalmic Epidemiol 15: 66-72.
50. Khandekar R, Al Raisi A (2008) Oman Eye Study 2005: validity of screening tests used in the glaucoma survey. East Mediterr Health J 14: 1360-1364.
51. Palimkar A, Khandekar R, Venkataraman V (2008) Prevalence and distribution of glaucoma in central India (Glaucoma Survey 2001). Indian J Ophthalmol 56: 57-62.
52. Vijaya L, George R, Baskaran M, Arvind H, Raju P, et al. (2008) Prevalence of primary open-angle glaucoma in an urban south Indian population and comparison with a rural population. The Chennai Glaucoma Study. Ophthalmology 115: 648-654.e1.
53. Zhang XL, Ren BC, He Y, Chen L, Sun NX, et al. (2008) Observation study on the relationship between the asymmetry of intraocular tension and glaucoma without previous diagnosis and treatment in Shaanxi rural people aged above 50. Guo Ji Yan Ke Za Zhi 8: 1194-1197.
54. Casson RJ, Marshall D, Newland HS, McGovern S, Muecke J, et al. (2009) Risk factors for early angle-closure disease in a Burmese population: the Meiktila Eye Study. Eye 23: 933-939.
55. Haq I, Khan Z, Khalique N, Amir A, Jilani FA, et al. (2009) Prevalence of common ocular morbidities in adult population of aligarh. Indian J Community Med 34: 195-201.
56. Wang S, Xu L, Jonas JB, Wong TY, Cui T, et al. Major eye diseases and risk factors associated with systemic hypertension in an adult Chinese population: the Beijing Eye Study. Ophthalmology 116: 2373-2380.
57. Garudadri C, Senthil S, Khanna RC, Sannapaneni K, Rao HB (2010) Prevalence and risk factors for primary glaucomas in adult urban and rural populations in the Andhra Pradesh Eye Disease Study. Ophthalmology 117: 1352-1359.
58. Huang TL, Hsu SY, Tsai RK, Sheu MM (2010) Etiology of ocular diseases in elderly Amis aborigines in Eastern Taiwan (The Amis Eye Study). Jpn J Ophthalmol 54: 266-271.
59. Nongpiur ME, Sakata LM, Friedman DS, He M, Chan YH, et al. (2010) Novel association of smaller anterior chamber width with angle closure in Singaporeans. Ophthalmology 117: 1967-1973.
60. Perera SA, Wong TY, Tay WT, Foster PJ, Saw SM, et al. (2010) Refractive error, axial dimensions, and primary open-angle glaucoma: the Singapore Malay Eye Study. Arch Ophthalmol 128: 900-905.
61. Sia DI, Edussuriya K, Sennanayake S, Senaratne T, Selva D, et al. (2010) Prevalence of and risk factors for primary open-angle glaucoma in central Sri Lanka: the Kandy eye study. Ophthalmic Epidemiol 17: 211-216.
62. Zheng Y, Wong TY, Lamoureux E, Mitchell P, Loon SC, et al. (2010) Diagnostic ability of Heidelberg Retina Tomography in detecting glaucoma in a population setting: the Singapore

Malay Eye Study. Ophthalmology 117: 290-297.
63. Zheng Y, Wong TY, Mitchell P, Friedman DS, He M, et al. (2010) Distribution of ocular perfusion pressure and its relationship with open-angle glaucoma: the singapore malay eye study. Invest Ophthalmol Vis Sci 51: 3399-3404.
64. Hsu WM (2011) Epidemiology of eye diseases among ethnic Chinese: data from the Shihpai eye study. J Exp Clin Med 3: 166-170.
65. Ishikawa M, Sawada Y, Sato N, Yoshitomi T (2011) Risk factors for primary open-angle glaucoma in Japanese subjects attending community health screenings. Clin Ophthalmol 5: 1531-1537.
66. Kim CS, Seong GJ, Lee NH, Song KC; Namil Study Group, Korean Glaucoma Society (2011) Prevalence of primary open-angle glaucoma in central South Korea the Namil study. Ophthalmology 118: 1024-1030.
67. Liang YB, Friedman DS, Zhou Q, Yang X, Sun LP, et al. (2011) Prevalence of primary open angle glaucoma in a rural adult Chinese population: the Handan eye study. Invest Ophthalmol Vis Sci 52: 8250-8257.
68. Thapa SS, Rana PP, Twayana SN, Shrestha MK, Paudel I, et al. (2011) Rationale, methods and baseline demographics of the Bhaktapur Glaucoma Study. Clin Experiment Ophthalmol 39: 126-134.
69. Wang D, Huang W, Li Y, Zheng Y, Foster PJ, et al. (2011) Intraocular pressure, central corneal thickness, and glaucoma in chinese adults: the liwan eye study. Am J Ophthalmol 152: 454-462.e1.
70. Yoon KC, Mun GH, Kim SD, Kim SH, Kim CY, et al. (2011) Prevalence of eye diseases in South Korea: data from the Korea National Health and Nutrition Examination Survey 2008-2009. Korean J Ophthalmol 25: 421-433.
71. Chung SD, Hu CC, Ho JD, Keller JJ, Wang TJ, et al. (2012) Open-angle glaucoma and the risk of erectile dysfunction: a population-based case-control study. Ophthalmology 119: 289-293.
72. Foo LL, Nongpiur ME, Allen JC, Perera SA, Friedman DS, et al. (2012) Determinants of angle width in Chinese Singaporeans. Ophthalmology 119: 278-282.
73. Kim M, Kim TW, Park KH, Kim JM (2012) Risk factors for primary open-angle glaucoma in South Korea: the Namil study. Jpn J Ophthalmol 56: 324-329.
74. Sng CC, Foo LL, Cheng CY, Allen JC Jr, He M, et al. (2012) Determinants of anterior chamber depth: the Singapore Chinese Eye Study. Ophthalmology 119: 1143-1150.
75. Suh W, Kee C; Namil Study Group and Korean Glaucoma Society (2012) The distribution of intraocular pressure in urban and in rural populations: the Namil study in South Korea. Am J Ophthalmol 154: 99-106.
76. Sun J, Zhou X, Kang Y, Yan L, Sun X, et al. (2012) Prevalence and risk factors for primary open-angle glaucoma in a rural northeast China population: a population-based survey in Bin County, Harbin. Eye 26: 283-291.
77. Wang YX, Hu LN, Yang H, Jonas JB, Xu L (2012) Frequency and associated factors of structural progression of open-angle glaucoma in the Beijing Eye Study. Br J Ophthalmol 96: 811-815.
78. Wang S, Xu L, Jonas JB, You QS, Wang YX, et al. (2012) Dyslipidemia and eye diseases in the adult Chinese population: the Beijing eye study. PLoS One 7: e26871.
79. Xu L, Jonas JB, Cui TT, You QS, Wang YX, et al. (2012) Beijing Eye Public Health Care Project. Ophthalmology 119: 1167-1174.
80. Zhou Q, Liang YB, Wong TY, Yang XH, Lian L, et al. (2012) Intraocular pressure and its

relationship to ocular and systemic factors in a healthy Chinese rural population: the Handan Eye Study. Ophthalmic Epidemiol 19: 278-284.
81. Choudhari NS, George R, Baskaran M, Ve RS, Raju P, et al. (2013) Can Intraocular Pressure Asymmetry Indicate Undiagnosed Primary Glaucoma? The Chennai Glaucoma Study. J Glaucoma 22: 31-35.
82. Katibeh M, Ziaei H, Pakravan M, Dehghan MH, Ramezani A, et al. (2013) The Yazd Eye Study-a population-based survey of adults aged 40-80 years: rationale, study design and baseline population data. Ophthalmic Epidemiol 20: 61-69.
83. Levkovitch-Verbin H Goldshtein, G Chodick, N Zigman, V Shalev (2013) The burden of glaucoma and its complications: a large population-based cohort study. Value Health 16: A176.
84. Pan CW, Cheung CY, Aung T, Cheung CM, Zheng YF, et al. (2013) Differential associations of myopia with major age-related eye diseases: the Singapore Indian Eye Study. Ophthalmology 120: 284-291.
85. Rauf A, Malik R, Bunce C, Wormald R (2013) The British Asian community eye study: outline of results on the prevalence of eye disease in British Asians with origins from the Indian subcontinent. Indian J Ophthalmol 61: 53-58.
86. Thapa SS, Poudyal I, Khanal S, Rens GV (2013) Results of the bhaktapur glaucoma study, Nepal. Nepal J Ophthalmol 5: 81-93.
87. Wang GQ, Bai ZX, Shi J, Luo S, Chang HF, et al. (2013) Prevalence and risk factors for eye diseases, blindness, and low vision in Lhasa, Tibet. Int J Ophthalmol 6: 237-241.
88. Wu R, Cheung CY, Saw SM, Mitchell P, Aung T, et al. (2013) Retinal vascular geometry and glaucoma: the Singapore Malay Eye Study. Ophthalmology 120: 77-83.
